# Experiences of Family Burden in Caring for the Severely Mentally Ill in a Foreign Land: A Qualitative Study of Chinese Immigrant Families in Toronto, Canada

**DOI:** 10.1177/13634615211000552

**Published:** 2021-03-23

**Authors:** Samuel Law, Lisa Andermann, Wendy Chow, Xing Wei Luo, Xiang Wang

**Affiliations:** 1 Community Mental Health Services, Mount Sinai Hospital, Canada; 2 Department of Psychiatry, University of Toronto, Canada; 3 Medical Psychological Institute, Second Xiangya Hospital of Central South University, P.R. China

**Keywords:** Burden, caretakers, Chinese, family, immigrants, serious mental illness

## Abstract

There is a relative dearth of qualitative studies on the actual experiences of families caring for members suffering from serious mental illness, and even less is known about disadvantaged ethnic minority immigrant families. This explorative qualitative study examines the burden experienced by 15 family members of Chinese immigrant background in Toronto, Canada. Six common themes emerged from the study: 1) significant worries about not being able to take care of ill members in the future; 2) on-going strain and changed family life; 3) pervasive social stigma, discrimination and lack of resources; 4) general appreciation of Canadian health and welfare systems and opportunities; 5) cultural factors and beliefs uniquely shape families’ support and caring commitment; and 6) families find various ways to cope and help themselves. Opportunities for improved care delivery based on these understandings are discussed.

## Introduction

In North America, family caregiver burden is on the rise, influenced by increasing life spans, smaller family sizes, decreases in long-term care availability, and increases in the complexity of responsibilities arising from technology and health system changes (Sales, 2003; Wirsén et al., 2017). Despite the reputation of “downward social spiral” with relationships, 83% of patients in North America with serious mental illness (SMI) such as schizophrenia have families living close by, maintain regular contact (80%), and many live with one or more family members (30–65%) (Lehman et al., 1998; Lehman & Steinwachs, 1998; Guarnaccia, 1998). In a large scale quantitative US study, Perlick et al., (2006) described four prominent components of burden found in family members caring for those with SMI: 1) difficult experience of patient problem behaviors; 2) patient impairment in activities of daily living; 3) perception of patient not being helpful; and 4) resource demands and disruptions in the caregiver’s personal routine. Their further analyses showed that ethno-racial background of being African American, when compared to Whites, was one of the strongest predictors of family burden, along with intensity of psychotic symptoms, presence of substance abuse, anti-social behavior, duration of illness, income, and education.

There is a limited number of studies on family burden that include factors such as ethnicity and immigration status. The National Alliance on Mental Illness (NAMI) Family-to-Family Education Program study found African Americans had higher levels of burden and negative caregiving experiences, and less knowledge of mental illness than their White counterparts (Smith et al., 2014). With mixed methods, Rosenfarb and colleagues (2006) found that families’ perceived burden of care affected rejecting attitudes towards their ill family members, and were more common among White caregivers than their Black counterparts. Magaña et al. (2007) found that Latino families in the US experienced comparatively higher level of burden than the general population, with significantly higher depressive symptoms, which were associated with caregivers’ young age, lower levels of education, and higher levels of the patients' mental illness symptoms. In general, studies found that immigrants, for a variety of reasons including a lack of access to resources, financial and language barriers, and culturally based beliefs, etc., are comparatively more involved in the caring of family members with SMI than the general population, with comparatively heightened personal costs (Awad & Voruganti, 2008; Breitborde, Lopez, Chang, Kopelowicz, & Zarate, 2009; Gater et al., 2014; Martens & Addington, 2001). Immigrant caretakers’ quality of life, financial stability, increased levels of stress, anxiety, and sense of stigmatization are negatively impacted (Carretero, Garces, Rodenas, & Sanjose, 2009; Papastavrou, Charalambous Tsangari & Karayiannis, 2010), while these groups were found to be at greater risk for mental illness and substance abuse disorders themselves (DeVylder & Lukens, 2013).

Among Chinese immigrants in North America, limited studies have also shown significant concerns. In their country of origin, with kinship-centred relational structures and limited community mental health services, over 90% of patients with schizophrenia live with their families, who are often the only source of support and care (Phillips, Pearson, Li, Xu, & Yang, 2002; Tang, Leung, & Lam, 2008). Immigrants to North America from this backdrop carry with them dramatically different caretaking understandings and caretaker identities. Compared to the general population, Chinese immigrants in North America are more likely to be involved in the routine caring of ill members, have delayed help-seeking, utilize fewer services, have more involuntary admissions and longer hospitalization stays, poorer adherence with treatment, higher relapse of illness, and lower rates of follow-up (Lin & Lin, 1981; Chiu, Lebenbaum Newman Zaheer & Kurdyak, 2016; Ziguras, Klimidis, Lewis, & Stuart, 2003; Klimidis, Hsiao, & Minas, 2007). Typical explanatory hypotheses include language barriers, shortage of culturally appropriate services, poor understanding and knowledge concerning mental illness, and stigma. (Blignault, Ponzio, Rong, & Eisenbruch, 2008; Chiu et al., 2016). In depth qualitative studies on Chinese immigrants’ caretaker burden are limited. A mixed methods study found cultural and immigration-related factors such as financial restraint, limited social interactions, stigma and shame related to illness, and various forms of household disruptions interacted with caregiver burden (Kung, 2003). One qualitative study of Chinese Canadian immigrant families coping with first episode psychosis found the families were more likely to hold more negative conceptions of mental illness, keep it secret from others, and blame the ill person for their illness relative to their Euro-Canadian counterparts (Ryder et al., 2000). In turn, these issues translated to reluctance and delay in seeking psychiatric treatment, more direct family care, and a higher level of family burden.

To broaden the understanding, this qualitative research aims to explore, in-depth, the lived experiences of families who care for family members with SMI, with particular attention to their prior experiences, narratives, values, expectations, and coping mechanisms. In turn, we hope to learn how to improve services for this growing, typically marginalized population.

## Methods

### Study overview

The main research question was: How do Chinese immigrant families understand and experience care giving when faced with socio-cultural and linguistic barriers in a new country? The development of the qualitative interview guide and data analysis were informed by constructivist grounded theory (Charmaz, 2014). In particular, the methodology helps to guide our research to explore the situations, actions, social psychological processes, and the social and cultural determinants related to the immigrant caretaking experience.

### Setting

This study was conducted at the Mount Sinai Hospital Assertive Community Treatment (ACT) team, located in downtown Toronto, Ontario. Chinese immigrants constitute about 11.1% of Canadian immigrants, and patients with schizophrenia tend to live with their family, with challenges of limited education, poor English skills, and high levels of stigma (Census, 2016; Chow et al, 2010; Chiu et al, 2016). This ACT team is unique as it serves specifically ethnic minorities with SMI (Yang, et al., 2005). About 40 of the 100 ACT patients are of Chinese background. Of these, about half have close family members involved in their care.

### Participants and recruitment

The inclusion criteria for family members were: 1) over 18 years of age; 2) having lived with the patient for more than a year; 3) patient has been part of the ACT team for six months or more; 4) suffering from no active mental illness; 5) of Chinese immigrant background (i.e. those born in another country and have become citizens or permanent residents; and 6) able to provide meaningful consent. The participants were recruited using a multi-stage process involving staff identifying the participants and Research Coordinator (RC) following up. Fifteen of the 18 family members approached agreed to participate. No obvious clinical and demographic (age, gender, income level) differences were noted between the participants and those who declined. Written consent was obtained from each patient and family member. Recruitment and interviews took place between 2013 and 2015. This study was approved by the Research Ethics Board at Mount Sinai Hospital.

### Data collection

Guided by literature review and input from the research team, a semi-structured qualitative interview guide was developed based on the validated Chinese version of the Family Burden Interview Schedule (FBIS) (Chien & Norman, 2004), originally developed by Pai and Kapur (1981). A semi-structured interviewing format allows an in-person, informal and open approach, yet guides the interview for known key areas of inquiry based on literature review and the main research question.

Family burden is a multifaceted subject often conceptually divided into subjective and objective dimensions (Hoenig & Hamilton, 1966; Braithwait, 1992; Schene, 1990). Subjective burden refers to a caregiver’s own perceptions of distress, stigma, worrying, shame, and guilt (Maurin & Boyd, 1990), alongside burden from grief and loss, chronic strain, and empathic pain (Marsh & Johnson, 1997). Objective burden includes more external and measurable dimensions of intensity of the illness, actual tasks of care, daily living and managing problems, changes of family routine, loss of leisure and employment, and impact on finances, time, and overall qualities of health and family life (Hjarthag, Helldin, Karilampi, & Norlander, 2010; Flyckt, Lothman, Jorgensen, Rylander, & Koernig, 2013, Maurin & Boyd, 1990).

The semi-structured interview aimed to capture both dimensions. A Master’s level RC, fluent in Mandarin and Cantonese conducted the interviews. All 15 interviews were concluded in a single session, either in a private setting in the community or at the ACT team office, in either Mandarin or Cantonese by the interviewee’s preference, on average lasting 60 minutes. The interviews began with open-ended questions to foster rapport building and reflection. The questions were designed to be broad enough to allow a wide range of experiences to be reported beyond the basic “sensitizing” dimensions, and emphasized learning the interviewees’ meanings and experiences. All interviews were audiotaped and transcribed in Chinese verbatim by the RC who carried out the interviews. We also collected sociodemographic data of the participants in person and related patient demographic and diagnostic information from a chart review.

### Data analysis

The RC checked for accuracy of the transcripts and then independently and sequentially open coded and wrote memos for the transcripts. To enhance cultural and linguistic interpretations of the information, a second analyst (SL) also reviewed the transcripts and made codes and memos. These codes and memos were then reviewed and discussed together to enhance the completeness and credibility of the analyses. Notes on the discussions were also taken to inform the ongoing coding process. The coding process was concurrent with the data collection as analyses performed during this time period were helpful to inform “sensitizing concepts” in later interviews. As analyses continued, analytic memos were generated to capture the key issues relevant to each code, and earlier transcripts were re-read and re-coded as necessary to capture newly emerging themes and concepts. The basic “sensitizing” concepts (e.g. financial impact, quality of life) found in the interview questions were used to inform initial thematic coding structure, while broader and novel concepts and themes were actively pursued. Frequent and salient codes were identified; thematically linked codes were clustered to aid the development of distinct and informative thematic categories. Data saturation was determined by the research team when no more new codes or categories emerged. In general, the two analysts were complementing each other and achieved the desired goals for completeness and gaining depth in the analyses. Disagreements were generally minor; when they occurred, consensus was reached through ongoing review and discussion. Quotations were selected to represent themes in the data, aiming to illustrate the thematic points beyond their specific content. The team met roughly every two to three months. All research team members except one are bilingual with fluency in English and Chinese.

## Results

### Participant demographic data

The participants (n = 15) included six mothers, four fathers, three husbands, one wife, and one daughter; their age ranged from 23–76. The patients (n = 11) were between the ages of 28–51 (M = 35.9; SD 7.3); they mainly suffered from schizophrenia and schizoaffective disorders (10/11, 90.9%); one person had severe Obsessive Compulsive Disorder. The length of time the patients have been on the ACT team ranged from 2–10 years (M = 5.5; SD = 2.3) (see Table 1).

**Table 1.** Clinical and demographic characteristics of study participants and family patient member.

Family member #; Manuscript ID	Family member relationship to patient; Age	Family member occupation	Family member education; English proficiency	Family years in Canada	Family Chinese regional origin	Family member immigration status	Patient age;Gender (M/F)	Patient diagnosis	Patient years with team
1; [M1]	Mother; 56	Homemaker	Middle school; Low	30	Southeast China (Fujian)	Permanent resident	30; Male	Schizoaffectivedisorder	4
2; [W1]	Wife; 30	Cashier	High school; Low	15	Southeast China(Fujian)	Permanent resident	30; Male	Schizoaffective disorder	4
3; [F1]	Father; 65	Factory labourer	High school; Low	13	Southeast China(Fujian)	Citizen	32; Male	Schizoaffective disorder	5
4; [M2]	Mother; 65	Factory labourer	High school; Low	13	Southeast China(Fujian)	Citizen	32; Male	Schizoaffective disorder	5
5; [B1]	Brother; 56	Real estate agent	University; High	20	Taiwan	Citizen	51; Female	Schizophrenia	6
6; [M3]	Mother; 68	Homemaker	Elementary school; Low	21	South China (Guangdong)	Citizen	37; Male	Schizophrenia	9
7; [M4]	Mother; 76	Small business owner	High school; Middle	25	South China (Guangdong)	Citizen	42; Female	Schizophrenia	10
8; [M5]	Mother; 60	High school teacher	University; Middle	11	East China (Shanghai)	Citizen	28; Male	Schizophrenia	5
9; [H1]	Husband; 48	Chinese medicine practitioner	University; Middle	6	East China(Jiangsu)	Citizen	42; Female	Schizophrenia	3
10; [M6]	Mother; 52	Homemaker	Elementary school; Low	20	South China (Guangdong)	Citizen	28; Male	OCD	6
11; [F2]	Father; 56	Factory labourer	High school; Low	20	South China(Guangdong)	Citizen	28; Male	OCD	6
12; [H2]	Husband; 35	Factory Labourer	Technical College; Low	12	Central China (unknown province)	Permanent resident	29; Female	Schizophrenia	2
13; [H3]	Husband; 38	Chinese medicine practitioner	University; Middle	13	East China (Shanghai)	Permanent resident	33; Female	Schizoaffective	4
14; [H4]	Husband; 49	Truck driver	Middle school; Low	13	South China (Guangdong)	Citizen	43; Female	Schizophrenia	7
15; [D1]	Daughter; 23	Office administrator	University; High	13	South China (Guangdong)	Citizen	43; Female	Schizophrenia	7

### Qualitative findings

#### Theme 1) Significant worries about not being able to take care of ill members in the future

##### Parent generation’s unrelenting concern

One mother [M2] put it simply when talking about her daughter: “*She is quite good right now. Stable. Only regret is no family. We are not going to be around for long. That’s a problem*.” Another parent [M3], worried about her own frailty, said: *“We two old ones cannot fall sick, if we fall sick, the child is finished, too.*” Another parent [F1] was also to the point: “*We are worried until we die. If we die, we won’t know how he is, then it is just that. We live. We worry. We just have to take care of him as long as we live. He is our son!*” Another mother [M1] captured the sentiments as follows: “*What I am most worried is how to take care of him in the future, when we are not around anymore!*”

##### Worry about lack of care is prevalent

Similarly, younger generation family members looking after their parents or siblings felt dread. One daughter [D1] of a patient said: “*Now I am worried about my youngest brother. When mom got sick, he was* very *young, he never got the good care; he is struggling now*. *How are we going to keep taking care of mom now?*”

#### Theme 2) Family caregivers experience on-going strain and changed family life

##### Burden of loss of hope

One parent [M1] summarized her experience as: “*When he was diagnosed it was the most difficult. It is incurable! That is the hardest. We cried every day for three years. Doctors said there is no cure. He was home for 10 years. People told us that we saved the government a lot of money by him staying at home.*” A husband who has been caring for his wife for over 20 years said: *“The illness has been so long, there is no more hope to recover*.”

##### Frequent worries of violence and stability

Along with hopelessness, parents worry about what their ill children’s behaviour. A mother [M3] lamented: *“No hope, if he can come quietly down for dinner, that’s already good and our best hope for peace…, I never know what he is up-to upstairs, I cannot sleep, I am worried.”* A father [F1] had a different kind of worry, with far reaching impact: “*We cannot leave him alone, when we come home the tables would be upside down and the home a mess. I go out to work and all I can think of is him staying at home, I cannot think of anything else. How can we feel at ease going out to work?”* And he [F1] added: “*He hits his mother, that’s the worst part about him, sometimes every two to four days… she is very afraid of him. Terrible.”*

##### Ongoing financial burden and worries

Financial worries are a prominent part of caregivers’ ‘unending’ worries. One parent [M3] said: *“Other parents are resting now, retired, but we have to keep working and care for him…, I am way over 60. I wish I can relax like other parents, go travel.”* Another parent [F2] echoed this: *“Because of him, we could not truly work for three years. Right now it is ok, we can borrow here and there. One day we may need to sell the house, but what can we do?”* One husband had to stay home: “*We are affected a lot, the biggest thing is our son is not finishing school. She has no way to care or help him when she herself is so sick…, I eventually stopped working driving long distance trucks to be at home.*” Other financial burden came from unexpected places. A husband recounted the stress when his wife lost the ability to care for their children: “*Children’s Aid came over. They would not allow the kids to be alone with her, so we had to use daycare. That cost so much, but Children’s Aid did not care how and where we send the kids, just not with her. We got into a lot of debt.”*

##### Relational and family life sacrifices and changes

Families also found it hard to manage their own relationships. One mother [M3] complained: *“I ask [patient] to come to church with me but [my husband] really went against that. He says don’t go. He takes him to casino and play cards and gamble…, and we fight over this.”* Another mother [M3] had very strong words about her marriage: *“If it wasn’t for our son, I would have left him tens of years ago. I don’t know how long I will last. We never help or console each other, he is a bastard*.” Home life was also trying for some. A daughter [D1] recalled: “*If I have to bring friends home. I usually don’t… I rarely did anyways…, my mom can be hostile for no reasons. I wanted to move out.*” Another mother [M2] recalled her home life changed quickly when her daughter needed help: “*When her husband left her, we just made a room for her in our house. We spend more time on Saturdays and Sundays with her. We looked after their daughter more. That’s what we did to make it work. That’s life.”* Another father [F2] echoed: “*We are always worried. He calls us anytime, saying this is not working, that is going wrong. Then we have to go and help him. We cannot be away. We used to go away here and there, but now we just stay around. We just stopped doing things for ourselves.*” As well, a husband captured their restricted life as: “*We had no social life, can’t leave children alone too long. Can’t go to work party, can’t visit friends. That was what became of us. In ten years, I only went back to China to see my parent once.”* The same husband also found the impact on their young children troubling: “*Kids used to ask, how come my mommy didn't come to pick me up like the other kids moms? The kids felt bad. Now they understand more.”* Strikingly, a mother [M6] made big life decisions based on her worry: “*I have this tumor and it’s been around four years. They recommended surgery and I said I could not. I have my son to care for. I cannot fall sick. The tumor is a little smaller now, I am watching it…”*

#### Theme 3) Pervasive social stigma, discrimination and lack of resources experienced as immigrants

##### Negative experiences of social judgments and discrimination

One mother [M5] recounted hardship dealing with her neighbours and family: “*My neighbours and relatives knew he has this illness, so they poke fun and laugh at him, and look down on us. They tell people that he is sick and no good, so we don’t have him come home to visit us much. They are really mean. We hardly interact now*.” A father [F2] recounted their dwindling social network: “*There are people who don’t understand mental illness, no idea about the medical part. They stopped coming to our house or being friends. They used to come when nothing is happening, now something has happened and they don't come anymore.”*

##### Rejections by family and relatives

One mother [M4] reported her experiences of rejection: “*My son’s mother-in-law was so against the relationship before they got married. It is about the schizophrenia, of course*.” A wife [W1] admitted that: “*When I learned that he has schizophrenia, I really thought about splitting up with him, but I stayed on, to stay in Canada, and he made my heart soft for him…*” She added: “*My parents really were against it. I was soft. Worried about genetics. Passing down to the kids’ generation. But we stuck it through.”* Stigma also impacted on social life. The same [W1] family member recalled her social isolation: “*I cannot talk to his mother or sister, they keep busy. I cannot even talk to my good friends because I don’t know what they will think of me… I just keep it to myself, keep it simple. It’s our business. No point broadcasting things… I do wish he had more friends.”* Another parent [F2] sorrowfully said: “*The illness is embarrassing to us. Some relatives know. Some we just avoid. Many of them just stopped asking. Maybe they look down on us.”*

##### Trying to manage stigma and discrimination in various difficult forms

Some families tried to rationalize. One father [F1] said: “*To be honest, and it does not sound good, but if he had cancer, he either lives or dies, but this kind of illness, what can we do? If it was cancer, we could detect early and save him, we could spend money and save him; with this illness, the whole family is game over. Cancer death is at least clear, this illness is life long and forever…what can you do, the entire family is dragged down, what can you do?*” Another parent [M5] invoked fate and fortune: “*No matter how you put it, it is very bad fortune to have this problem [of mental illness]. We are doomed as a family*.” One other parent [M1] told her story of avoidance and isolation: “*I cannot, and I am afraid of telling other people or relatives. Other people don't know and don't understand this illness. If we did he will never have a girlfriend. We were all alone.*”

##### Lack of resources

Getting psychiatric help was very difficult for many, one mother [M6] described the ordeal: “*They had meetings and interviews and then meetings to decide. I was so stressed, not certain whether they will take my son. I had so much hope for some good help. He has been to so many doctors. … I could not sleep well and my health took a hit. I fainted four times.”*

#### Theme 4) General appreciation of Canadian health and welfare systems and opportunities

##### Thankful for disability welfare and public psychiatric care

Without any prompt, one mother [M4] said: *“The Canadian medical system is very good here, really. We could not have afforded the medications and hospitalizations in China. He was beat up by co-patients there a lot. I had to live in the hospital to accompany him. Here there are single rooms and good nursing protection. We would have been bankrupted if we were still in China…”* One mother [M2] also said: “*The government assistance is so helpful; it relieved our burden. Our lives changed for the better as otherwise we would not be able to pay for her [treatment]. Canada has such good social benefits.”* Another family member echoed: “*She takes medications that are so expensive. If the government steps away, we will not be able to make do. Too much burden*.” One husband [H2] put things in perspective despite adversity: “*The society does a pretty good job supporting and coordinating for us. You cannot do it alone. Even the Children’s Aid helped our kids a lot, and the ODSP [Ontario Disability Support Program]… there is good social structure and welfare, just not many friends and family.”*

##### Appreciation for Canadian culture of acceptance and immigration opportunities

One mother [M4] said: *“It’s different in China. The village people would laugh at us for this kind of illness, even close family relatives… here the social workers, big shots in the community, the doctors, they care and take care of him. People are respectful. No looking down on us. So lucky we are here.”* Also finding relief in Canada, a family member [W1] recounted: “*In China people like to talk. This one talks to that one, rumors abound. We don't want to go back, what for? People like to talk and lots of prejudice against his mental illness. An uncle of mine sent his daughter-in-law home [terminating an arranged marriage] after she had a mental breakdown. They are like that.*” Similar sentiments were coming from a husband: “*In Canada the government is attentive to her. Poor people’s lives are not bad… In China mental health patients are more discriminated [against], that happens. They think you are always a patient. In Canada they think this is a brain illness. In China they think you are off your mind. They see you coming and they walk far away to avoid you.”* Speaking in appreciation of his immigrant opportunity, one father [F1] said: “*We came with nothing, but now we all own houses, but [the patient] will never, though.”*

#### Theme 5) Cultural beliefs and immigration factors uniquely shape families’ support and caring commitment

##### Strong notions of obligation, loyalty and sense of responsibility

A father [F1] talked of parental obligation: *“You cannot count on siblings or others to look after [the patient], only parents can, or a wife. If no wife, he will be not cared for, and [we parents] can only be with him as long as we live, but it is not forever for him.”* One parent [M3] talked about the importance of progeny: *“He had a wife who left after four years and unable to bear a child… there was no life security without kids…, we don’t blame her for leaving.”* Another parent [M4] added: *“Siblings can help, but they have their families, their mortgages, their car loans, their kids. We don’t blame his sister. We had no choice because we were his parents, we had to protect him on our own.”* One mother [M1] put it simply: “*We all feel we need and should help him. It makes a big difference having a family around.”* Another mother [M5] summarized her traditional cultural views: “*He likes me to visit every week, I cannot go away much. I take care of him until someday when I cannot do it anymore. I don’t know what that day is. I am getting old, I worry. I wish he had someone in his life to help him. I think like a traditional Chinese.”*

##### Cultural notions of filial piety and gender role stereotyping

A family member used a Chinese idiom to describe a parent’s resentment of a child with SMI: “*There is a Chinese saying that children with long term illness are not going to be filial kids because they cannot take care of their parents. …. The parents get tired and exhausted over time. ‘We have spent so much heart and energy, why are you still not normal?’”*^1^ On the other hand, children struggle to fulfill their filial duties and other cultural traditions, as a daughter [D1] complained: “*My four siblings are talking [about] how to split the work. May be even send mom to an institution. But we cannot agree on this. The logic of thinking is, ‘you are the daughter, you are supposed to be cooking and cleaning’… My grandmother kind of trained me to take on the responsibility. You know. I guess being the second and being a girl. It’s more like it’s just my responsibility. The son is always pampered. She [grandmother] didn’t put any burden on my older brother. She put all the responsibility on me.*” This respondent did not only shoulder extraordinary caring duties from a young age, but also felt she could not refuse the demands and expectations of the family due to a sense of filial duty and culturally based practices cultivated by her family because she was female.

##### Lament on loss of cultural support and resources

Coming from a close knit, traditional cultural background, one family tried to secure support through matrimonial match-making for their son, and the father [F1] reported: *“We borrowed a lot of money to try to find him a wife in China. Still owe a lot. Still no wife. Things are very expensive, but what can we do, this must be done.”* Living in urban Canada is often a challenge, the same father [F1] also said: “*It’s just bad luck when a family is hit with an illness like this. Family connections are watered down, just less here than in the village in China where we came from* – *we lived close to each other, all relatives lived in the same village; here maybe one lives in Scarborough, one in Mississauga [suburbs of Toronto], way too far; our village was 600 square metres, 8,000 people, I could walk the entire place in half an hour.*” Another family member [M3] recalled: “*In my old village the women typically did not work, they come and go and visit a lot… Here women all work, same as the men. They live far apart, no one to help. I regret immigrating here.”* One husband summarized succinctly: “*The good thing about China is you have lots of friends and relatives, they can chip in and help. Here you have no one; that is a big advantage back home.”*

#### Theme 6) Families find various ways to cope and help themselves

##### Drawing strengths from within and being an ally and advocate in treatment

One mother [M2] credited her own family strength: “*We had no problem with her illness. We all knew this is a serious illness. Our family all supported each other. When she is well, we are well. When she is sick, we feel sick, lots of worries*.” One family member [D1] found her own principles in life helpful (*“If life is hard, then you need to try harder. That’s the only way out.”*) while maintaining hope through finishing school*: “Education helped me growing up, having my own job, and trying to cope with this situation. It’s still hard to get that education with troubles that we have. It kind of provides me that sense of security…, it opens doors where you can get a job.”* A mother [M5] echoed: *“I always think studying more and getting more education helped me, it gives me a sense of security, so I can be a better care-taker.”* One family member simply cherished his role as a supporter of treatment after a protracted time of medication refusal in his wife: “*I keep telling her, if you take your medication, you are just normal like the rest of us. We can manage that…”*

##### Strengths through spirituality and acculturation

Others drew support from outside and through acculturation, as one mother [M3] recounted: *“Let me tell you, my biggest fortune is that I joined a church, it has given me the most strength – the strength to handle his illness; otherwise I would have collapsed long time ago. You follow the local culture and see what helps you. He likes going to church, people are nice to him there; no one bullies or takes advantage of him there.”* Another mother [M5] echoed: “*He could not tell who is nice and who is bad, but he became a Christian and the pastor is very kind and nice to him. He really trusts him and enjoys going to church for some activities when he could. When the pastor is busy, he told my son to visit him still. My life is given to me by God.”*

##### Strengths in pride as caretaker

Being proud of their care and commitment towards their ill members was also a source of strength. One parent [M3] said: *“Other people may look down on him, but we are family, we cannot do that. If there is no one sick, then there will be no need for hospitals. Sickness is normal. We look after him like family should, we need to protect him.”* Another mother [M5] reported with pride: *“I have brought him up from a tiny baby. Don’t mind me saying so, if other families have a sick child, they won’t care for him as much as I do; I spoil him too much.”*

##### Strengths in acceptance and understanding of mental illness

One family member [W1] found acceptance helpful: *“I treat him like a normal person, chat with him, argue a bit; if I always think he is ill and need special treatment I will be very tired. I don’t put* too *much in my mind, and don’t hold grudges for long. If I do, I will be exhausted. No one wants to have someone who is ill. You have to deal with it and move on, don’t put it in your mind forever.”* Others have found strength simply through better understanding, as one husband said: “*In the beginning we had no understanding, now understanding can help. The only hope is she continues treatment. Now understanding is the best help for her treatment.”*

##### Strengths in seeking community of mutual support and pride of independence

Some also found encouragement in each other. One mother [M1] recalled: “*When I went to a family support meeting for the first time, I saw a lot of unhappy people, but I was feeling relieved. I started to know I was not alone.*” In a different domain, one family [M4] adapted in somewhat unusual ways: *“We don’t want government support. We didn’t have much money because he is sick, so we picked beer bottles, making 30 or 40 dollars a day that would be enough to live on.”* Another family member ventured on how they manage financially: “*Some people here in Canada will spend all their check and live nicely for the first half of the month. Then money is all gone. We are fine. We never let money run out. Less money, you just spend less. Simple.”*

## Discussion

This qualitative study of Chinese immigrant families’ experiences in caring for family members with SMI identified a number of themes. To start, participants studied here share many similarities with other immigrants in terms of experiencing lower social economic status, language barriers, social isolation, and a lack of understanding of and access to social resources (Kokanovic, Petersen, & Klimidis, 2006; Kirmayer, 1989).

One strong theme found in the study is the culturally shaped extraordinary commitment in the caretaking of their family members. Chinese cultural heritage generally places enormous value on the family and sees it as the building block of a healthy society, and families are expected to take on the responsibility of caring for its members. In turn, the family’s caring ability reflects the health and respectability of the family (Poon & Harvey, 2013; Hong & Domokos-Cheng Ham, 2000; Wong, Tsui, Pearson, Chen, & Chiu, 2004). These ideals may be generally perceived as positive, but can also negatively affect both the patient, in their feeling of having failed to achieve normalcy, and the care giver, in their feeling of having inadequately cared for the ill (Hsiao, Klimidis, Minas, & Tan, 2006; Sun, 2014).

Socio-economic reasons may also play a role. Historically and currently in China, there have been very low levels of formal, government-run community based support for families, and the cost of psychiatric care in hospitals is increasingly prohibitive, reinforcing the need for self-reliance in family caring (Zhou et al., 2016; Zhai, Guo, Chen, Zhao, & Su, 2013). When Chinese immigrate abroad, these cultural aspects most likely continue to shape the way they manage family members with mental illnesses. Wu and Tseng (1985) identified several important cultural continuation elements integral to Chinese communities worldwide, among them: family based collective responsibility, emphasis on the parent-child relationship and bond, value of social interaction, preference for emotional control and morality-based reasoning, and valuation of education and achievement are most prominent. From this perspective, severe psychotic mental illness disrupts all the valued cultural aspects and the family is often left with a desperate sense that it needs to take the primary responsibility to cope and restore the lost bonds, social interactions, and respectable achievements. Burden is more likely to take root when compared to a more Western model where mental illness is perceived as requiring medical or psychiatric attention, not something to be resolved by the family (Ryder et al., 2000).

More specifically, immigrant parents of elderly age are particularly noted in the current study to bear a high level of worry and uncertainty about how to take care of their ill children in the future. This may partly reflect a context in which accessing adequate and appropriate care is still perceived to be difficult, and comfort level with the current services is limited. These sentiments from the immigrants are notably similar to the findings in Asia where public services are less robust. For example, researchers in Taiwan (Hou, Ke, Su, Lung, & Huang, 2008) have reported similar worries regarding patients’ illness, dependency, and concerns about their safety and illness relapses. As well, researchers in Hong Kong (Chien, Chan, & Morrissey, 2007) and China (Li, Lambert, & Lambert, 2007; Yang, Bryne, & Chiu, 2016) have found older parents prominently worried about the future, compounded by perceived lack of social support, low income, poorer quality of life, and low number of family members living with patients. There are also research findings that show older caregivers tend to have higher burden levels than younger ones (Vella & Pai, 2012), possibly related to their health conditions and looming financial precarity (Grover, Avasthi, Chakrabarti, Bhansali, & Kulhara, 2005). It is also likely a reflection of traditional Chinese culture where the elderly in a family are to be respected as the ‘heads of household’ with responsibility to take care of all family members. Failure to meet this responsibility, particularly when immigration related loss or lowering of employment, loss or lowered availability and control of resources (and therefore respectability) is often associated with a sense of guilt and shame, and it may shape the expressions of worry about the future of loved ones (Wong et al., 2004; Lin & Lin, 1981).

The current study’s findings on the plight of burden also echoes research on role captivity and role overload, when caretakers are facing inordinate responsibilities and feeling trapped within (Pearlin, 1983). Echoing similar studies, many participants in our sample felt their lives were inescapably altered or diminished as they had to resign to putting their ill family members’ needs before their own (Ostman & Kjellin, 2002; Van Der Voort, Goossens, & Van Der Bij, 2009; Suro & Weisman de Maman, 2013). Relatedly, one should be aware that when immigrant families like the ones studied here are highly committed to assisting their ill family members, it may easily lead to an underestimation of the resources needed in the community, or the need to inform them of available resources. As well, care services may need to pay attention to immigrant patients’ level of dependence, and ability to be independent as part of assessments and recovery goal setting.

A few participants have put extraordinary effort into securing their ill family members with a life partner through marriage. This effort likely reflects a strong cultural expectation for marriage as a form of domestic support and continuation of family. It also gives context to the extended worry and grief found in families’ beliefs that the ill member will be less capable of caring for themselves in the long run (Richardson, Cobham, Murray, & McDermott, 2011). An appreciation of this culturally based imperative is helpful for health care providers to understand the clients’ priority in familial goals, hope, and attendant sense of fulfillment or burden. International research finds this also common in Latino and Indian cultures (Hernandez & Barrio, 2015; Chakrabarti, 2010).

The current study also found a high level of disruption of the participants’ normal lives and social activities. A recent large family burden study in an acute in-patient setting in China found social isolation and disruption of caregiver routines very problematic (He, Zhou, Sun, Guo, & Rosenheck, 2015; Zhou et al., 2016). As immigrants, in a setting where social networks and support are already diminished, this is likely even more impactful. Designing and developing care to provide relief, respite, and social support for immigrant families appears highly worthwhile.

Furthermore, the current study found that the effects of stigma on mental illness is pernicious on many levels. Stigma led some families to shun social activities, isolate themselves, hide the illness, and avoid seeking help. Notably, stigma acts as a wedge for these families who celebrate close knit social network of support and family interdependence, yet face shame and reluctance to communicate or obtain support for mental illness from their extended families. This retreat and high reliance on the nuclear family likely have contributed to their caretaker burden over time (Phillips et al., 2002; Poon, & Harvey, 2013). These findings echo other research on impact of stigma in Chinese communities such as delayed help seeking, and less adherence with treatment (Poon & Harvey, 2013; Ryder et al., 2000; Hsu, 1985; Chiu, Poon, Fong, & Tsoh, 2000; Mak & Cheung, 2008).

Beyond discrimination and social stigma, family participants in the current study also described an internalized sense of shame, guilt and regret that carried deep invisible scars (Kung, 2003; Larson & Corrigan 2008). This is particularly impactful for those who hold the belief that their neglect caused or worsened the development of mental illness in their family members, a not uncommon explanatory model for the cause of schizophrenia in the Chinese community (Phillips, Li, Stroup, & Xin, 2000; Furnham & Chan, 2004; Hsiao et al., 2006). Service providers would do well in gaining understanding of the illness explanatory models from both patients and their families, so psychoeducation and culturally meaningful care could be better targeted. Furthermore, practices and policies could focus more on relieving such internalized blame (Chiu, Wei, & Lee, 2006).

The current study also identified a mixed experiences of lacking understanding of, access to, and perceived satisfaction with mental health services. Immigrant families are typically under-served by the general mental health system (e.g. Kokanovic et al., 2006). It is worth noting that evidence shows ethno-cultural services that target marginalized ethnic minorities do significantly improve clinical outcomes, ranging from children (Yeh, Takeuchi, & Su, 1994), to the chronically mentally ill (Yang et al., 2005). Proper and adequate services are essential to ameliorate delayed help seeking, poor attendance for follow up and low adherence with treatment (Ryder et al., 2000).

On a different note, there is a very clear appreciation of the welfare system from the current study’s participants. This is an honest and grateful positive recognition that is not typically reported by other research. The Canadian system should take some pride and note that a well-funded welfare and health system should not go unrecognized, and its continuation and improvement as part of a basic social safety net is essential to reach those who are the hardest to reach.

Lastly, the current study found a strong theme in that family caretakers have developed various ways to cope with their worries and burden. Drawing strength and solace from religious beliefs and spiritual support seemed very helpful to a number of participants. Acceptance of the illness helped with coping for a number of others. Chinese culture may have some predisposition to appreciating that some major life events happen due to larger than life forces that are beyond one’s control, and one can only accept and cope with the outcome (Wong et al., 2004). This cultural aspect may be very helpful in considering newer psychological counselling models such as Acceptance and Commitment Therapy where a focus on acceptance is emphasized, and promotes finding new meaning and purpose in the caring journey for the family members (Öst, 2014). Whether the coping is through spirituality, acceptance, humour, or taking pride in their care, the participants have arrived at these gains in a manner reflective of a path of recovery, where they have learned to maintain hope, accept their challenges, empower themselves over time, and find new meaning in what they have achieved – parallel to the recovery journey of the patients themselves (Anthony, Cohen, Farkas, & Gagne, 2002; Myers, 2010; Slade, Phelan, & Thornicroft, 1998). More could be done. A recent meta-analysis on interventions to improve caretaker experiences found psychoeducation, family support groups, and problem-solving bibliotherapy to be positive, though the quality of the evidence was low (Yesufu-Udechuku, Harrison, & Mayo-Wilson, 2015). More effort to include these components to support any family, not the least marginalized immigrant families, is an area demanding more active support (Dixon et al., 2014).

### Limitations

The current study has a number of limitations. The sample size is relatively small, the age range wide, and comes from one service program. We recognize that Chinese immigrants are diverse in socioeconomic and geographic and ethnic origins, and our sample’s representativeness is limited. It is hoped that the current findings reflect some of the general experiences of immigrants who have struggled with similar burdens. There may also be some selection bias as not all families approached agreed to participate. The general impression of those who participated and declined were not remarkably different. We also did not have enough sample size for sub-analyses, such as how different relationships to patients may influence the sense of burden. Other limitations may also be related to the sensitive topic itself. Some participants may not be open to talk about burden as it may seem they are complaining about something they should be doing and therefore risk losing face (Hsiao et al., 2006). Additional limitations may be related to potential biases as the participants were introduced by team workers and participants may be predisposed to talking about more positive aspects of their experiences.

Finally, despite basing our semi-structured interview on a validated Chinese instrument on caretaker burden, the participants reflected experiences that are not all captured by the notion of burden, as they included unreserved commitment, hope and adaptation, acceptance, and pride in their work. The notion of “burden” may not be the best fit for everyone. It may have implied some negative connotation, an aversive label, suggesting something unwanted, negative, invariably imposed upon, when some family members may not consider giving care a burden, but rather an obligation that is willingly honoured as part of a cherished family role (Sales, 2003), and something positive and rewarding that helped to add purpose and positive meaning in life (Chen & Greenberg, 2004).

### Implications for practice

Our study has identified several important caretaker burdens and issues for the Chinese immigrant families. Having service providers paying particular attention to parent or elderly caretakers’ worries would be a strong recommendation for all service providers. Determining and understanding the special coping strategies of the family caretakers and helping to strengthen these and their social and support network in general would also be synergistically productive and rewarding for both caretakers and service providers. The need to combat illness stigma at the patient, family and societal levels are again highlighted. Last but not the least, on-going collaboration with the family system and assessing their needs and experiences should be part of the standard of care working with chronic and marginalized populations.

## Note

1 The idiom, “jiǔ bìng wú xiào zǐ” (久病无孝子), describes parents who have chronic illness tend to have unfilial children because of the long-term burden and deprivation exacted on the children, causing them to be less filial over time. The participant here used it in a reversed sense.
